# Novel Mitochondria-Targeted Furocoumarin Derivatives as Possible Anti-Cancer Agents

**DOI:** 10.3389/fonc.2018.00122

**Published:** 2018-04-23

**Authors:** Andrea Mattarei, Matteo Romio, Antonella Managò, Mario Zoratti, Cristina Paradisi, Ildikò Szabò, Luigi Leanza, Lucia Biasutto

**Affiliations:** ^1^Department of Chemical Sciences, University of Padova, Padova, Italy; ^2^Department of Biology, University of Padova, Padova, Italy; ^3^CNR Neuroscience Institute, Padova, Italy; ^4^Department of Biomedical Sciences, University of Padova, Padova, Italy

**Keywords:** Kv1.3, mitochondria-targeting, cancer, triphenylphosphonium, psoralens, pancreatic duct adenocarcinoma

## Abstract

Targeting small molecules to appropriate subcellular compartments is a way to increase their selectivity and effectiveness while minimizing side effects. This can be accomplished either by stably incorporating specific “homing” properties into the structure of the active principle, or by attaching to it a targeting moiety *via* a labile linker, i.e., by producing a “targeting pro-drug.” Mitochondria are a recognized therapeutic target in oncology, and blocking the population of the potassium channel Kv1.3 residing in the inner mitochondrial membrane (mtKv1.3) has been shown to cause apoptosis of cancerous cells expressing it. These concepts have led us to devise novel, mitochondria-targeted, membrane-permeant drug candidates containing the furocoumarin (psoralenic) ring system and the triphenylphosphonium (TPP) lipophilic cation. The strategy has proven effective in various cancer models, including pancreatic ductal adenocarcinoma, melanoma, and glioblastoma, stimulating us to devise further novel molecules to extend and diversify the range of available drugs of this type. New compounds were synthesized and tested *in vitro*; one of them—a prodrug in which the coumarinic moiety and the TPP group are linked by a bridge comprising a labile carbonate bond system—proved quite effective in *in vitro* cytotoxicity assays. Selective death induction is attributed to inhibition of mtKv1.3. This results in oxidative stress, which is fatal for the already-stressed malignant cells. This compound may thus be a candidate drug for the mtKv1.3-targeting therapeutic approach.

## Introduction

In therapeutic oncology, the ultimate goal is to cause the death of all cancerous and cancer stem cells while inflicting negligible damage to healthy cells and organs. Several strategies have been adopted in an effort to achieve this difficult result. One is to selectively hit only the unwanted cells with an effective death-inducing treatment. Examples of this approach include focused radiation therapy, the many attempts at delivering drugs selectively to tumoral cells, and immunotherapy. Another is aiming a drug at a molecular target which is expressed specifically by cancer cells and/or whose function is more cogently needed by cancer cells than by healthy ones, including of course malfunctioning oncogenes. An example may be the development of small molecule inhibitors of deregulated oncogenic kinases. A third one may be to exploit an intrinsic characteristic of cancer cells, such as increased aerobic glycolysis (the Warburg effect) or their rapid growth and the associated redox stress they experience (1). This latter feature is of particular interest for this paper. An elevated production of ROS is a characteristic of rapidly proliferating cells (2). In cancerous cells, it is induced by various mechanisms (1). For example, K-ras hyperactivity has been reported to lead to suppression of respiratory chain (RC) complex-I, ROS generation, mitochondrial dysfunction, and switch to glycolytic metabolism (3, 4). Tumor suppressor p53 on the other hand acts *via* Bcl-2 family proteins to reduce mitochondrial ROS, and its loss or malfunction may thus lead to an increase in their production (5, 6). In fact, anti-apoptotic proteins of the Bcl-2 family, such as Bcl-XL and Bcl-w, reportedly increase mt ROS production. They do this by binding and neutralizing Bax, which reduces ROS by interacting with complex I (7, 8).

This stressed state can be exploited to induce cell death (1, 9–11). Excessive ROS can cause cell death by processes such as apoptosis (12) [mediated, for example, by redox-sensitive apoptosis signal-regulating kinase family members (13)], necrosis (14), and ferroptosis (15). An oxidative stress exceeding the “death threshold” can be achieved either by weakening the cellular antioxidant defenses which keep it within “safe” limits (16)—for example, by inhibiting a member of the peroxiredoxin system (17, 18)—or by increasing it. In turn, this latter option can be achieved either by using drugs which are themselves redox-active [e.g., Q-7BTPI (19)], or by stimulating the cells’ ROS-producing apparatuses, such as the mitochondrial RC [e.g., Ref. (20)].

Given their key role in cancer metabolism, progression, and survival (9, 21–26) and in apoptosis, mitochondria are a focus of anti-cancer chemotherapy (27–29). Of relevance here, mitochondrial ion channels are potential targets of strategies aiming to stress cancer cells to death. They influence mitochondrial membrane potential ΔΨ, ROS production, volume, and ion homeostasis (30). Pharmacological manipulation of mitochondrial ion channels can lead to cell death bypassing the upstream players of intrinsic apoptosis (p53 status, Bax/Bak/Bcl-2 expression and alterations of cytosolic signaling pathways) (31).

**Figure 1:**
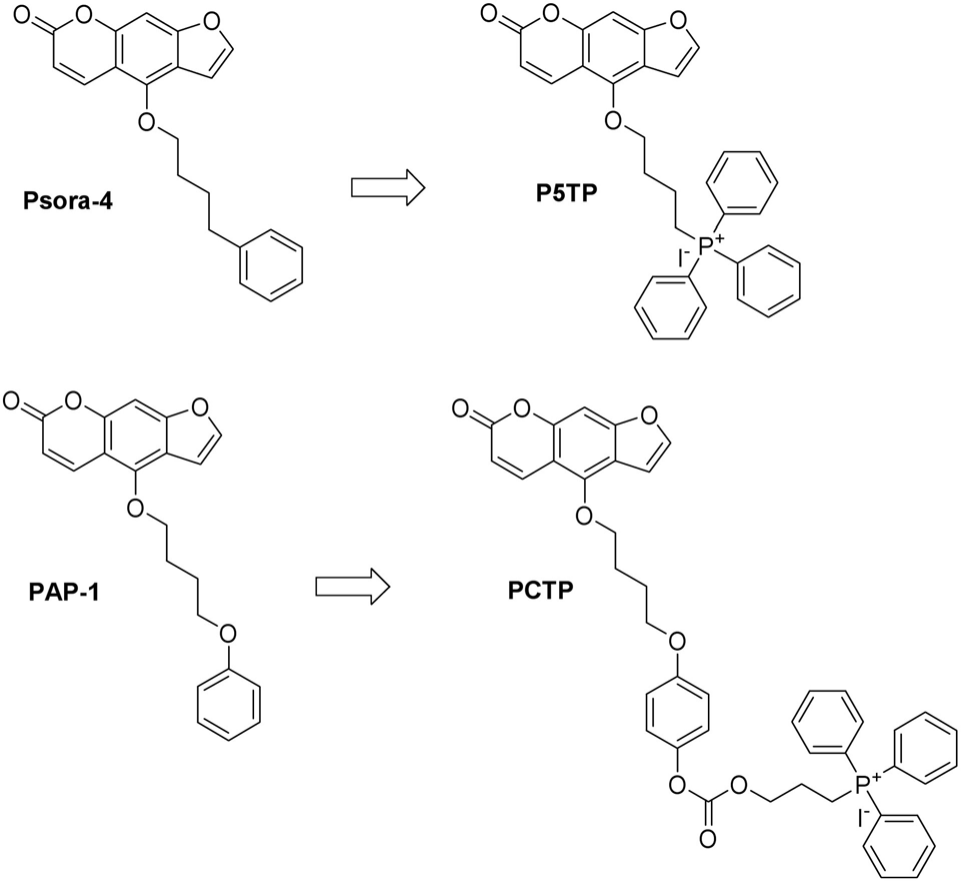
Chemical structures of the compounds studied in this work and their precursors.

In particular, our group has uncovered (32, 33) a crucial role of mitochondrial potassium-selective channel mtKv1.3 blockage by pro-apoptotic Bax in the apoptotic death of cells expressing mtKv1.3, which include many cancer cell lines (34). The other finding this line of research descends from is the observation (35) that 5-(4-phenylbutoxy)psoralen (Psora-4) (Figure 1), a membrane-permeant molecule, blocked Kv1.3 with an EC_50_ of 3 nM. A derivative, PAP-1, was less effective but more selective for Kv1.3 vs Kv1.5, which is also often expressed in the mitochondria of cancer cells. We used these compounds to show that pharmacological inhibition of mtKv1.3 could cause the same outcome as inhibition by Bax, i.e., death by apoptosis (36, 37). This outcome is currently understood to result from the following chain of events: stopping the depolarizing K^+^ influx causes inner mitochondrial membrane (IMM) hyperpolarization, with ensuing increased ROS level, activation of the mitochondrial permeability transition pore, mitochondrial swelling, loss of transmembrane potential, loss of cytochrome c, and further ROS release (36).

5-(4-phenylbutoxy)psoralen and PAP-1, however, had only a modest effect on cancerous cells when used at pharmacologically meaningful concentrations. To improve their effectiveness we sought to target the drugs to the IMM and mitochondrial matrix. The most effective and popular strategy for mitochondrial targeting relies on conjugating the drug to a lipophilic, membrane-permeant cation, most often triphenylphosphonium (TPP) (38–41). Various drugs based on this design and producing cytotoxic oxidative stress in cancerous cells have already been produced: mitochondria-targeted vitamin E succinate (MitoVES) (42–44), a construct interacting with RC complex-II; MitoMets (45, 46), metformin derivatives inhibiting RC complex I and inducing ROS production; MitoTam (47), based on tamoxifen and likewise acting *via* RC complex-I; mitochondriotropic derivatives of the polyphenols resveratrol (20, 48) and quercetin (19, 49), also causing deadly redox stress in cultured cells *via* the RC or concentration-enhanced autoxidation, respectively. We thus synthesized two TPP-comprising PAP-1 derivatives, PAPTP and PCARBTP, both of which turned out to be promising chemotherapeutic agents, selectively eliminating cancerous cells *in vitro* and in *in vivo* oncological models, including orthotopic melanoma and pancreatic ductal adenocarcinoma (PDAC) (50). These compounds caused the death of pathological cells independently of the expression levels of key pro- or anti-apoptosis proteins, such as Bax, Bak, Bcl-2, or p53. Importantly, they had no significant impact on healthy tissues and cells, including the immune system of mice and humans (50). The structure of PCARBTP combines two concepts: mitochondrial targeting, conferred by TPP, and prodrug function, provided by the carbamate link connecting the two parts of the molecule, PAP-1 and TPP. Since the carbamate group is hydrolyzed over several minutes in a physiological environment, this device allows the delivery of the essentially unmodified active agent to mitochondria.

Given the promising results, we have extended the search for anti-tumoral agents combining the Kv1.3-inhibiting furocoumarin structure and the mitochondriotropic TPP group.

## Materials and Methods

### Chemistry

Details of the synthetic procedures used in this study are provided in the Supplementary Material.

### Cell Lines

B16F10 cells (ATCC) were grown in Minimum Essential Media (MEM, Thermo Fisher Scientific) supplemented with 10 mM HEPES buffer (pH 7.4), 10% (v/v) fetal bovine serum (FBS), 100 U/mL penicillin G, 0.1 mg/mL streptomycin, and 1% non-essential amino acids (100× solution; Thermo Fisher Scientific). Lymphocytes (Jurkat, CTLL-2, and K562) were grown in RPMI 1640 (Thermo Fisher Scientific), supplemented as MEM. Medium for CTLL-2 was further supplemented with four units/mL/day of mouse interleukin-2 (IL-2). A panel of pancreatic cancer cell lines (51) was used: BxPC3, AsPC1, Capan-1, and PANC-1. BxPC3 derived from the body of the pancreas of a patient with adenocarcinoma. These cells are not prone to give metastasis and they are poorly differentiated. The other three lines were originally obtained from metastases (AsPC-1, Capan-1) or have considerable metastatic potential (PANC-1). All were provided by ATCC. AsPC1 and BxPC3 were cultured in RPMI-1640 supplemented with 10% FBS “GOLD” (PAA Laboratories/GE Healthcare Life Sciences), 1 mM GlutaMAX, and 1 mM sodium pyruvate (Thermo Fisher Scientific). PANC-1 were cultured in DMEM (4.5 g/L d-glucose) supplemented with 10% FBS “GOLD”, 1 mM GlutaMAX and 1 mM sodium pyruvate. Capan-1 cells were grown in IMEM supplemented with 20% FBS “GOLD,” 1 mM GlutaMAX, and 1 mM sodium pyruvate. The HPV16-E6E7—immortalized human pancreatic duct epithelial cells (HPDE), kindly provided by Dr. Ming-Sound Tsao (Ontario Cancer Institute, Toronto, ON, Canada) (52) were used as a model for benign pancreatic ductal epithelium. The complete HPDE growth medium was a mixture of 50% RPMI 1640, supplemented with 10% FCS and 1 mM GlutaMAX, and 50% keratinocyte medium SFM (Thermo Fisher Scientific) supplemented with 0.025% bovine pituitary extract, 2.5 mg/L epidermal growth factor (Thermo Fisher Scientific).

### Cell Viability and Cell Death Assays

For cell growth/viability MTT assays we used a protocol previously described (36, 50). Briefly, cells were seeded (5–10 × 10^3^ cells/well) in standard 96-well plates and allowed to grow in medium (200 µL) for 24 h to ensure attachment. The growth medium was then replaced in the dark with a medium that contained the desired compound (from a stock solution in DMSO) at the final concentration. The final concentration of DMSO was 0.1% or lower in all cases (including controls). To inhibit multiple drug resistance (MDR) “pumps,” where indicated we used non-toxic concentrations of cyclosporine H (CSH) (1 µM; Sequoia) and Probenecid (100 µM; Sigma Aldrich) in the case of CTLL-2 cells, and of CSH only (4 µM) for the other cell lines. After incubation for 24 h, CellTiter 96 AQUEOUS One solution (Promega, Italy) was added to each well as indicated by the supplier. Absorbance was measured at 490 nm to detect formazan formation using a Packard Spectra Count 96-well plate reader.

For cell death assays of non-adherent cells, cells were incubated with the test substances for 24 h, washed in HBSS, and resuspended in DMEM without serum and Phenol Red and incubated for 30 min at 37°C in the dark with Annexin-V FLUOS (Roche) (1 µL/200 μL sample). DMSO concentration was <0.1% in all cases. Flow cytometry analysis was carried out after the labeling period with a Becton Dickinson FACS Canto II flow cytometer and data were processed by quadrant statistics using BD VISTA software.

### Downregulation of Kv1.3 Expression by siRNA

The sequences for the siRNA targeting human Kv1.3 were coupled to Alexa Fluo 555 (Hs_KCNA3_1 Flexi tube siRNA for Kv1.3 and All-star negative control siRNA as scramble/control; Qiagen). Jurkat cells were transfected by electroporation, as previously reported (36). After 48 h from transfection, cells were treated for 24 h with the various compounds as indicated. Cell death was then evaluated by the binding of fluorescein isothiocyanate (FITC)-labeled Annexin-V and FACS analysis.

### Mitochondrial Morphology, Membrane Potential, and ROS Production

Mitochondrial morphology was studied in melanoma B16F10 cells. 1 × 10^5^ cells were seeded in a 6-well plate with 2 mL of complete medium. After 24 h medium was replaced with 1 mL HBSS supplemented with 500 nM Mitotracker green (Thermo Scientific). Cells were incubated at 37°C in the dark for 20 min and then the mitochondrial network was observed by confocal microscopy using a Leica DMI6000 fluorescence microscope with confocal settings (Leica Microsystem, Wetzalar, Germany).

Mitochondrial membrane potential and ROS production were measured in leukemic Jurkat T cells. 5 × 10^5^ cells were resuspended in 300 µL of HBSS supplemented either with 20 nM TMRM or 1 µM MitoSOX. Cells were incubated for 20 min at 37°C in the dark. Then, cells were diluted by the addition of further 1.2 mL of HBSS and analyzed by FACS (FACSanto II, Beckton Dickson).

### Western Blot

Kv1.3 protein expression was assessed after transfection with control (“scramble”) and anti-Kv1.3 siRNA. Jurkat cells from parallel siRNA experiments were lysed overnight refrigeration at −80°C in lysis buffer (25 mM Tris pH 7.8 + 2.5 mM EDTA + 10% glycerol + 1% NP-40 + 2 mM DTT). After thawing, debris was centrifuged off at 20,000 × *g* for 10 min at 4°C. Supernatants were collected and protein concentration was determined using the BCA method in a 96-well plate (200 μL total volume for each well) incubating at 37°C in the dark for 30 min. Absorbance at 540 nm was measured by a Packard Spectra Count 96-well plate reader. Proteins were separated by SDS-PAGE in a 10% polyacrylamide gel. After separation by electrophoresis, gels were blotted overnight at 4°C onto polyvinylidene fluoride membranes and then membranes were blocked with a 10% solution of defatted milk and were incubated with the following primary antibodies overnight at 4°C: anti-Kv1.3 (1:200, rabbit polyclonal, Alomone Labs APC-101); anti-GAPDH (1:1,000, mouse monoclonal, Millipore MAB374). After washing, the membranes were developed using corresponding anti-mouse or anti-rabbit secondary antibodies (Calbiochem). The antibody signal was detected with enhanced chemiluminescence substrate (SuperSignal West Pico Chemiluminescent Substrate, Thermo Scientific).

### Statistics

Statistical significance of the effects was assessed by paired *t*-test or two-way ANOVA analysis.

## Results and Discussion

One of the concepts we tested was to minimize the changes to the structure of the “parent” drug, Psora-4, while still turning it into a mitochondria-targeted drug. Thus, we simply substituted the distal phenyl ring with the TPP group (P5TP, Figure 1).

A second approach was that of attaching a mitochondria-targeting group to PAP-1. This strategy had proved successful when attaching the TPP moiety *via* a stable bond system or *via* a labile linker comprising a carbamate group (50). We thus tested another labile “joint,” the carbonate group, producing PCTP (Figure 1).

The rationale for the preparation of these new derivatives was based on previous studies which underlined the importance of not altering the planar furocoumarin system. The modification at position five of the psoralen scaffold did not affect the ability of the molecule to block the potassium cation inside the cavity of the channel as proposed by Zimin and coworkers (53).

For both derivatives the synthesis started with the natural compound bergapten (5-methoxypsoralen) and utilized the key intermediate PSBI (Figure S1 in Supplementary Material). The conversion of bergapten to PSBI was achieved by demethylation to bergaptol (2) promoted by BBr_3_, followed by alkylation with 1-bromo-4-chlorobutane to obtain the chloro-intermediate 3 and substitution of chloride with iodide *via* the Finkelstein reaction. Details are provided in Supplementary Material.

PCTP underwent a slow hydrolysis in DMEM, at pH 7.4 and 37°C, yielding PAP-OH and 4-TPP-butan-1-ol iodide with a *t*_1/2_ of about 17 h (data not shown).

**Figure 2:**
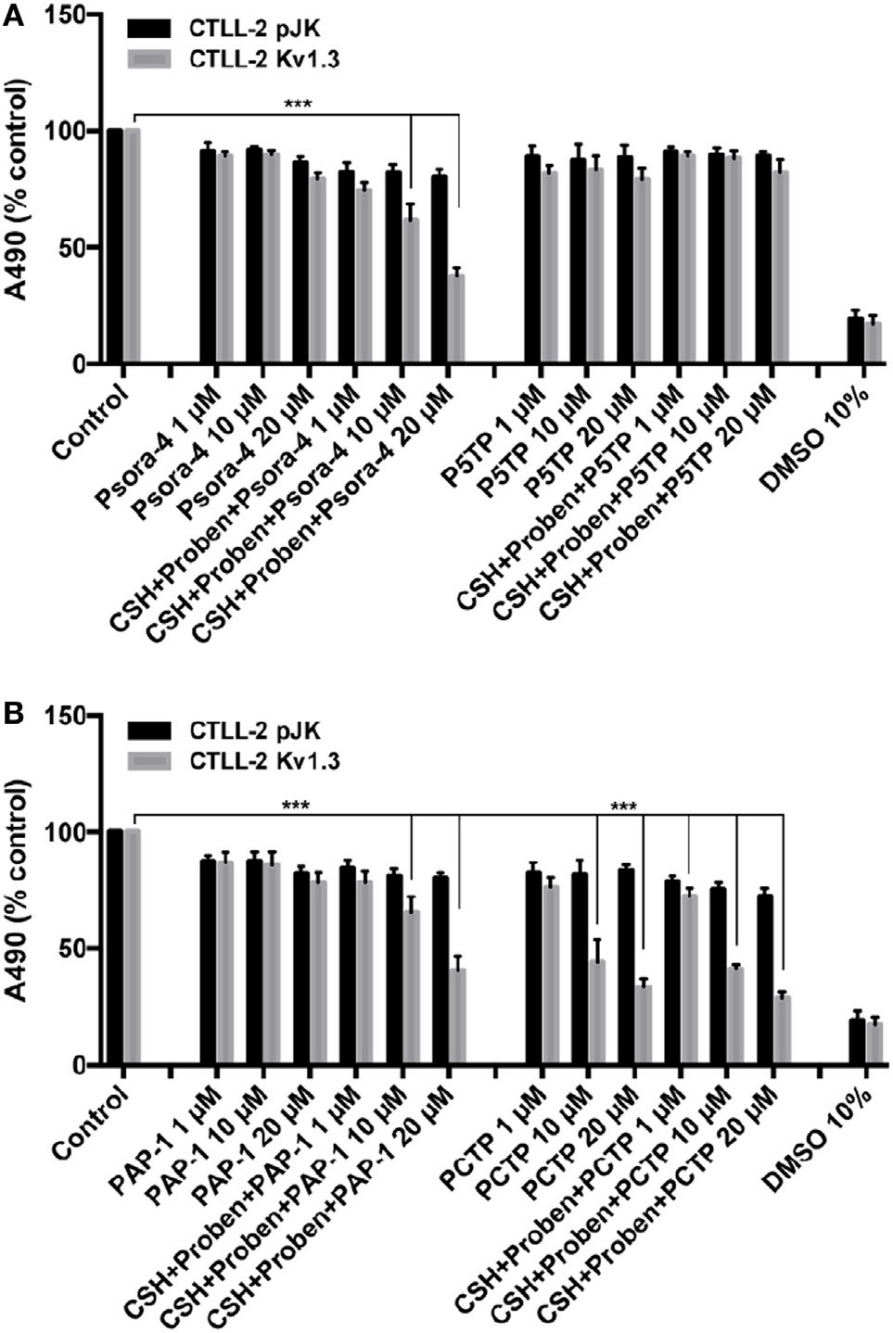
PCTP selectively eliminates Kv1.3-expressing cells. Murine lymphocyte CTLL-2 cells were transfected either with the empty vector (CTLL-2 pJK) or with the expression vector for Kv1.3 (CTLL-2 Kv1.3). Cell viability was assessed by MTT assay after 24 h of incubation with Psora-4, P5TP **(A)**, PAP-1, or PCTP **(B)** at different concentrations either without or with the addition of 1 µM cyclosporine H and 100 µM probenecid as multidrug resistance pumps inhibitors. DMSO 10% was used as positive control, since it is toxic at this concentration. Results are reported as mean percentage of viable cells normalized with respect to untreated cells (*n* = 3; ***p < 0.001).

The new derivatives were first screened for their cytotoxic activity on murine CTLL-2 lymphocytes. These cells do not express Kv1.3, and were either transfected with an empty vector to provide a control (CTLL-2/pJK) or stably transfected with an expression vector for Kv1.3 (CTLL-2/Kv1.3) (54) (Figure 2). Cell survival was assessed with the MTT assay. P5TP did not represent an improvement over Psora-4 or PAP-1 (Figure 2A). On the other hand, PCTP proved remarkably effective and exhibited selectivity toward cells expressing Kv1.3: while viability of CTLL-2/Kv1.3 cells decreased in a dose-dependent manner, that of CTLL-2/pJK cells was only slightly affected (Figure 2B).

Since MDR pumps may play a crucial role in extruding drugs from cells, MTT assays were also performed in the presence of MDR inhibitors (cyclosporin H and probenecid). In this specific case, MDR inhibition sensitized CTLL-2/Kv1.3 cells to Psora-4 and PAP-1, as previously reported (36), while there were no differences in the activity observed with PCTP. This observation suggests that the positively charged compound may escape MDR action due to a rapid “electrophoretic” transport through the plasma membrane, due to the negative-inside electrical potential difference. This has been already proposed for mitoVES. Contrary to VES, mitoVES was not a substrate for the ABCA1 pump in non-small cell lung carcinoma H1299 cells (55).

**Figure 3:**
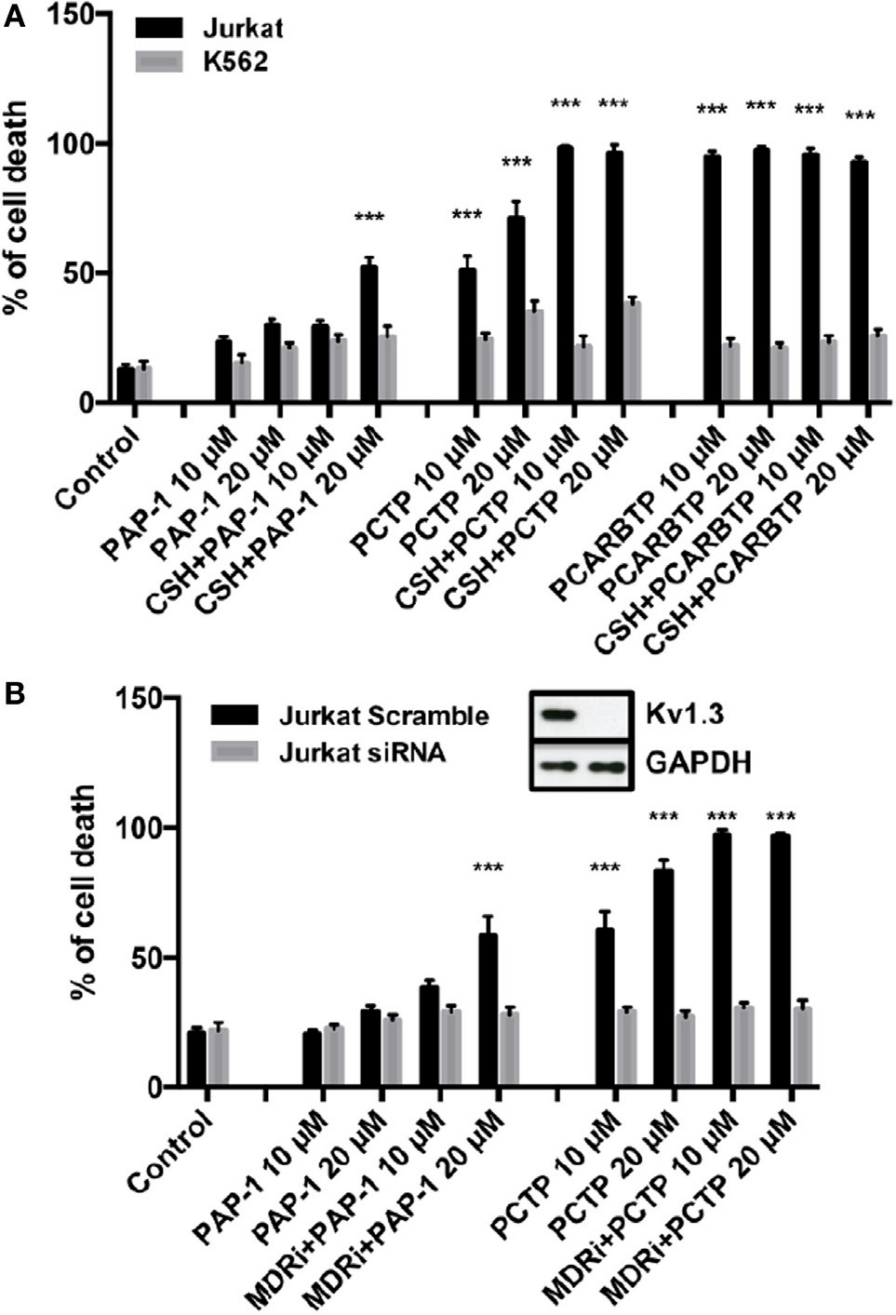
PCTP is specific in inducing cell death by Kv1.3 inhibition. **(A)** Jurkat T lymphocytes and leukemic K562 cells were treated for 24 h with PAP-1, PCTP, or PCARBTP at the indicated concentrations with or without the addition of 4 µM cyclosporine H as multidrug resistance pumps inhibitor. Cell death by apoptosis was then determined by incubation with fluorescein isothiocyanate-labeled annexin-V for 20 min at 37°C in the dark. Annexin-V positive cells were measured by FACS analysis (*n* = 3; ***p < 0.001 vs control). **(B)** Jurkat cells were either transfected with a control siRNA (Scramble) or siRNA against Kv1.3 (siRNA) and after 48 h from the transfection they were treated as in **(A)** with PAP-1 and PCTP (*n* = 3; ***p < 0.001 vs control). Insert: Kv1.3 downregulation was assessed by Western blot after siRNA transfection. A representative image is shown of three independent observations. GAPDH was used as loading control.

We then tested PCTP on two human leukemic cell lines: Jurkat leukemia T cells and K562 chronic myelogenous leukemia cells (Figure 3A). Cell death was determined by Annexin-V-FITC staining and FACS analysis. As expected, PCTP induced apoptosis only in Kv1.3-expressing Jurkat cells (54), while it was quite ineffective in killing K562 cells, which lack Kv1.3 (34, 56).

To further demonstrate Kv1.3 involvement in apoptosis induction by PCTP, Jurkat cells were transiently transfected with siRNA targeting Kv1.3 to reduce its expression (36). These cells have the peculiarity that they express only Kv1.3 among the potassium channels of the Kv family (54). Experiments confirmed that Kv1.3 expression is crucial for cell death induction by PCTP, since Kv1.3 silencing protected the cells from death (Figure 3B).

**Figure 4:**
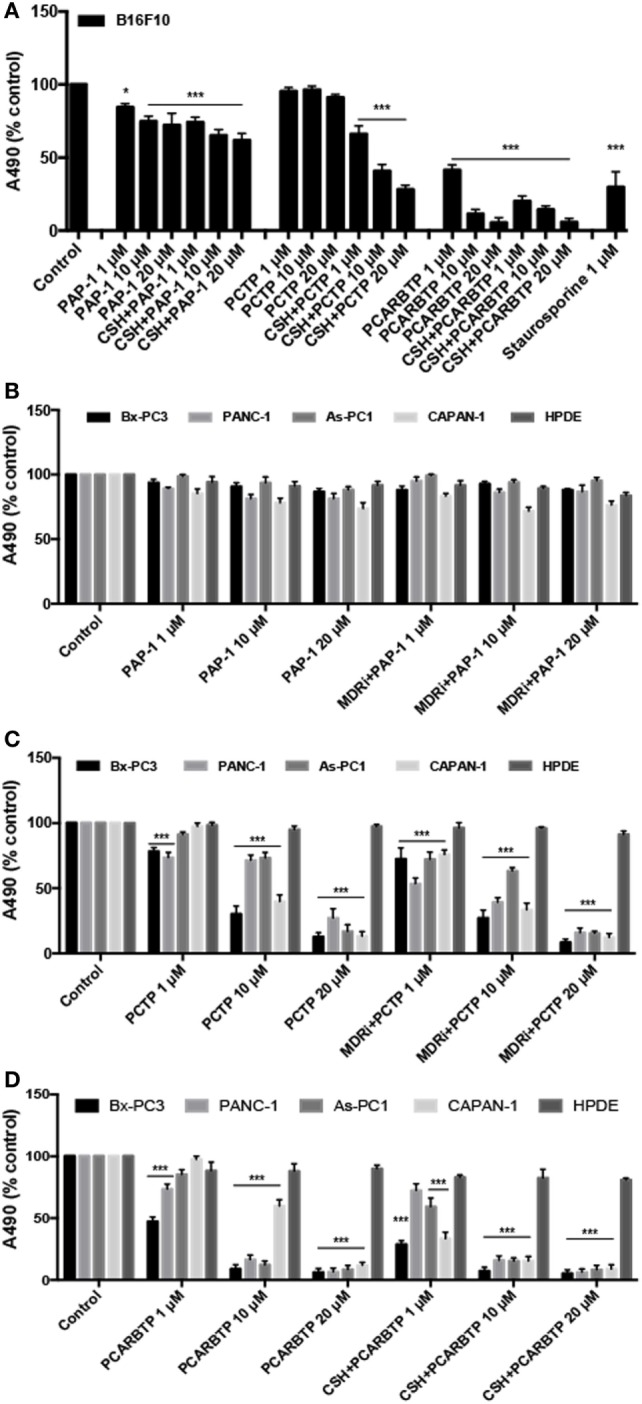
PCTP reduces the viability of Kv1.3-expressing cells. **(A)** Mouse melanoma B16F10 cells were treated either with PAP-1, PCTP, or PCARBTP for 24 h, with or without 4 µM cyclosporine H (CSH) as multidrug resistance pumps inhibitor. Results are reported as mean percentage of viable cells normalized with respect to untreated cells (*n* = 3; *p < 0.05; ***p < 0.001, vs control). **(B,C)** Four human pancreatic ductal adenocarcinoma cell lines (Bx-PC3, PANC-1, As-PC1, CAPAN-1) and a non tumoral human pancreatic duct epithelial line (HPDE, used as a negative control) were treated for 24 h with PAP-1 **(B)**, PCTP **(C)**, or PCARBTP **(D)**, both in absence or presence of 4 µM CSH. Mean percentage of viable cells normalized with respect to untreated cells (*n* = 3; ***p < 0.001 vs control).

We proceeded testing PCTP also with other Kv1.3-expressing cancer cell lines. We took advantage of a mouse B16F10 melanoma cell line (Figure 4A), which also expresses Kv1.3 in mitochondria, as we have shown before (36). In this case the presence of MDR inhibitors was crucial, as already observed with PAP-1. Nevertheless, PCTP is more powerful than the precursor in triggering cell death.

The derivative was finally tested on various PDAC cell lines, which have been shown to express Kv1.3 (51). All have been previously characterized, and were found to be mutated in p53, to express variable but robust levels of Bcl2-family anti-apoptotic proteins and to be largely resistant to standard chemotherapeutics (57–59). Most of them (with exception of Bx PC-3 cells) are also mutated in K-ras (57). These cell lines provide an *in vitro* model of one of the most feared and untreatable human cancers, for which a viable pharmacological approach is much needed.

Interestingly, also in this case PCTP proved remarkably effective in inducing cell death (Figure 4C) while its precursor, PAP-1, was essentially inactive (Figure 4B). Cytotoxicity varied somewhat from cell line to cell line (Figure 4C). PCTP was confirmed to induce apoptosis (not shown). These results again show that mitochondriotropic mitoKv1.3 inhibitors can overcome chemoresistance, exerting their cytotoxic effects despite alterations of the cellular anti-apoptotic apparatus.

**Figure 5:**
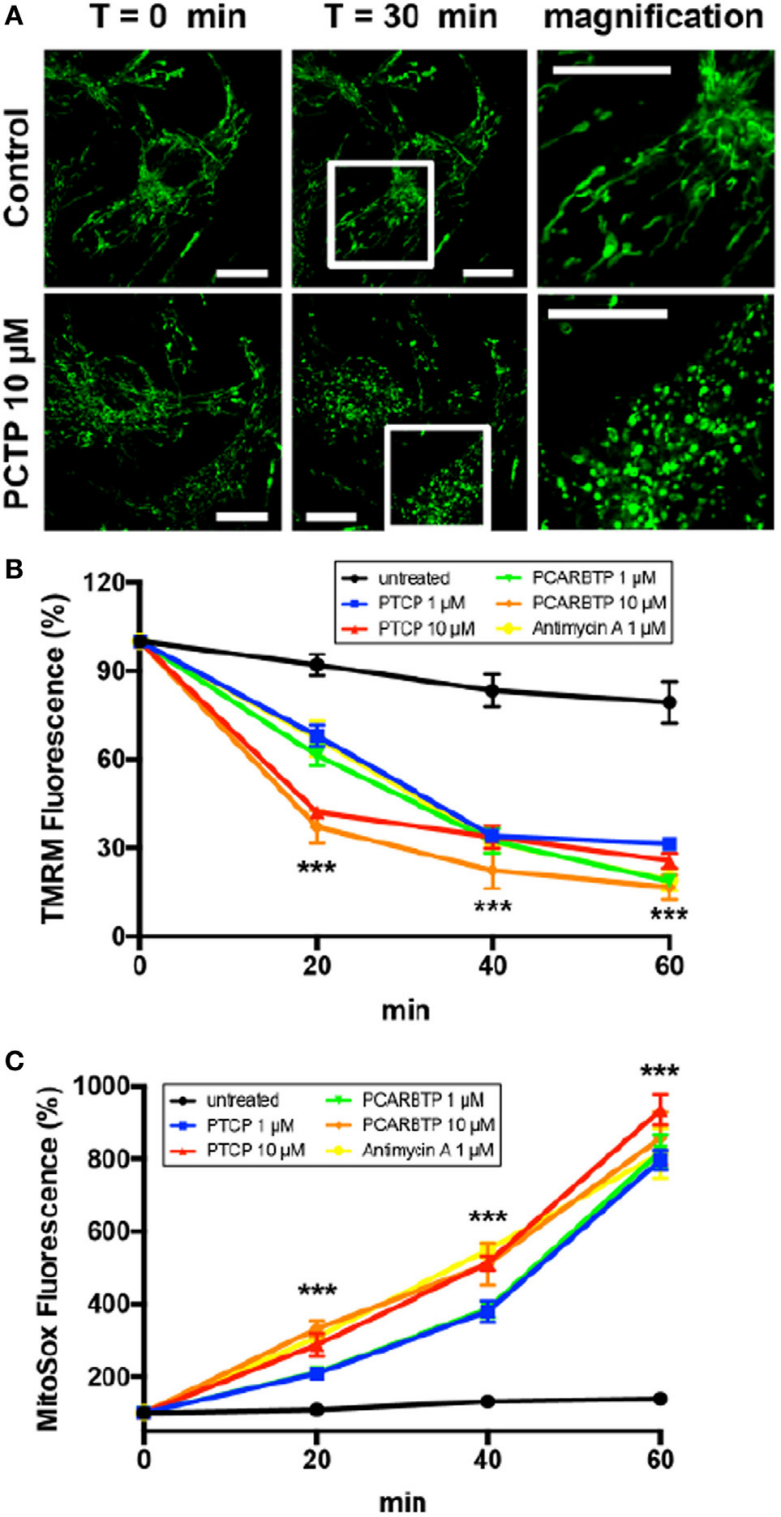
PCTP induces mitochondrial swelling, decrease in membrane potential, and increase in ROS production in Kv1.3-expressing cells. **(A)** Mitochondrial morphology was observed in B16F10 cells after staining the mitochondrial network by incubation for 20 min at 37°C with 500 nM Mitotracker green. The effects on mitochondria have been observed by confocal microscopy after 30 min of incubation either with or without 10 µM PCTP. The images are representative of three independent experiments (*n* = 3; Bars = 15 µm). **(B)** Mitochondrial membrane potential was measured by FACS analysis of TMRM fluorescence in leukemic Jurkat T cells. Values are reported as percentage of the initial fluorescence (*n* = 3; ***p < 0.001 with respect to untreated, all other conditions). **(C)** Mitochondrial ROS production was measured by FACS analysis of the increase in the fluorescence of MitoSOX in leukemic Jurkat T cells. Values are reported as percentage of the initial fluorescence (*n* = 3; ***p < 0.001 with respect to untreated, all other conditions).

We investigated the impact of PCTP on the mitochondria of intact cells. To monitor morphological changes we used B16F10 cells labeled with the permanent mitochondrial marker MitoTracker Green (Figure 5A). The exemplary images in Figure 5A show that the mitochondrial network underwent fragmentation. Mitochondrial fission has been firmly associated with the process of apoptosis (60–62). Mitochondrial depolarization (TMRM staining), and ROS generation (MitoSOX™ Red staining) were observed using Jurkat cells in FACS experiments (Figures 5B,C, respectively).

These observations are fully coherent with the mechanistic model deduced from the data obtained studying apoptosis (32) and using PAPTP to induce it (50): the initial event is channel inhibition, with consequent production of ROS. In turn, ROS promote the onset of the permeability transition, resulting in mitochondrial depolarization and further ROS release. The effects of PCTP on cancerous cells *in vitro* are comparable to those of PAPT and PCARBTP [Figures 4 and 5; (50)]. PCTP might conceivably even outperform these latter compounds *in vivo*, depending on factors such as pharmacokinetics and the rate of hydrolysis of the carbonate bond system. The mitochondrial effects suggest that the compound might have significant undesirable effects on healthy cells. It is, however, of relevance that non-tumoral, fast-growing, Kv1.3-expressing HPDE (51, 63) were not affected by PCTP (Figure 4C), as was the case also for PCARBTP (Figure 4D). While *in vivo* work is needed to investigate this crucial point, this observation suggests that PCTP might resemble PAPTP and PCARBTP in acting specifically on cancerous cells, sparing others.

## Conclusion

After PCARB, we have identified another mitochondriotropic prodrug of PAP-OH, PCTP, with marked pro-apoptotic effects on Kv1.3-expressing cancerous cells, including four PDAC lines. As is also the case for other mitochondriotropic psoralenic derivatives, its administration *in vitro* causes mitochondrial dysfunction and ROS generation. The results definitely warrant further testing in *in vivo* oncological models.

## Author Contributions

AMat, MR, AMan, LB, and LL performed experiments. AMat, LL, IS, MZ, and CP designed research. LL, IS, MZ, and CP analyzed results. LL, LB, MZ, AMat, and IS wrote the article.

## Conflict of Interest Statement

The authors declare that the research was conducted in the absence of any commercial or financial relationships that could be construed as a potential conflict of interest. The reviewer VC declared a shared affiliation, with no collaboration, with the authors to the handling Editor.
