# Supplementary material for: Novel Mitochondria-Targeted Furocoumarin Derivatives as Possible Anti-Cancer Agents
Source: Front Oncol. 2018 Apr 23;8:122. doi: 10.3389/fonc.2018.00122 (PMC5925966; doi:10.3389/fonc.2018.00122)
Supplement: Supplementary file 1 [file Presentation_1.PDF]

Novel mitochondria-targeted furocoumarin derivatives as possible anti-cancer agents

Andrea Mattarei[1], Matteo Romio[1], Antonella Managò[2], Mario Zoratti[3,4], Cristina Paradisi[1], Ildikò Szabò[2], Luigi Leanza[2]*, Lucia Biasutto[3,4]*

[1] Dept. Chemical Sciences, University of Padova, Padova, Italy
[2] Dept. Biology, University of Padova, Padova, Italy
[3] CNR Neuroscience Institute, Padova, Italy
[4] Dept. Biomedical Sciences, University of Padova, Padova, Italy

Supplementary Data

Material and Methods

Chemistry

Starting materials and reagents were purchased from Sigma-Aldrich, TCI, Fluka, Riedel-de Haen (Seelze, Germany), Prolabo (Fonyenay sous Bois, France), Carbosynth (Compton, Berckshire, UK), and were used as received. ^{1}H-NMR and ^{13}C-NMR spectra were recorded with a Bruker AC 250F spectrometer (operating at 250 MHz for ^{1}HNMR or at 62.9 MHz for ^{13}C-NMR), with a Bruker 300 UltraShield spectrometer (operating at 300 MHz for ^{1}H-NMR or at 75 MHz for ^{13}C-NMR), or with a Bruker 500 UltraShield spectrometer (operating at 500 MHz for ^{1}H-NMR or at 126 MHz for ^{13}C-NMR). Chemical shifts (δ) are given in ppm, and the residual solvent signal was used as an internal standard. TLCs were run on silica gel supported on plastic (Macherey-Nagel Polygram®SIL G/UV254, silica thickness 0.2 mm) and were visualized by UV detection. Flash chromatography was performed on silica gel (Macherey-Nagel 60, 230-400 mesh granulometry (0.063-0.040 mm)) under air pressure. The solvents were of analytical or synthetic grade and were used without further purification. Fluorescence/UV-Vis spectra were recorded at 25°C with a Perkin-Elmer LS-55 spectrofluorimeter. Quartz cells with an optical path length of 1 cm were used for measurement of both absorption and fluorescence.

Synthesis

Figure S1. Synthesis of PSBI, the common key intermediate for the synthesis of P5TP and PCTP. Reagents and conditions: i) BBr_3 in DCM, rt, 100 min, N_2, quantitative; ii) 1-bromo-4-chlorobutane, Cs_2CO_3, DMF, 50°C, overnight, N_2, 93%; iii) NaI, acetone, reflux, 24 h, quantitative.

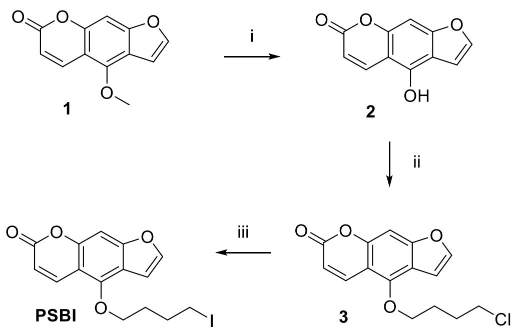

4-Hydroxy-7H-furo-[3,2-g]benzopiran-7-one, **[2]**. A BBr_3 solution (1 M in DCM, 10 mmol, 5.0 eq) was slowly added at room temperature and under nitrogen to a stirred bergapten (2.0 mmol, 1 eq) solution in anhydrous dichloromethane (20 mL). After 100 minutes, the mixture was washed with saturated aqueous $NaHCO_3$ (100 mL) and extracted with ethyl acetate (3 × 300 mL). The combined

organic layers were dried over $MgSO_4$ and the solvent was removed under vacuum to obtain **2** as an off–white solid (100% yield). ^{1}H-NMR (250 MHz, DMSO-d_6): δ = 11.31 (br, 1H), 8.25 (d, J = 9.7 Hz, 1H), 7.90 (d, J = 2.1 Hz, 1H), 7.19 (d, J = 2.9 Hz, 1H), 7.15 (s, 1H), 6.25 (d, J = 9.7 Hz, 1H) ppm; ^{13}C-NMR (62.9 MHz, DMSO-d_6): δ = 160.4, 157.0, 152.6, 147.9, 144.7, 139.6, 112.5, 110.7, 104.8, 103.7, 90.8 ppm; ESI-MS (ion trap): m/z: 203 $[M+H]^+$.

4-(4-clorobutoxy)-7H-furo-[3,2-g]chromen-7-one, **[3]**: Compound **2** (3.5 mmol, 1.0 eq in 25 mL), Cs_2CO_3 (5.2 mmol, 1.5 eq) and 1-bromo-4-chlorobutane (5.2 mmol, 1.5 eq) were suspended in anhydrous DMF (25 mL) and stirred under inert atmosphere at 50°C overnight. After this time, ethyl acetate (100 mL) was added and the mixture was extracted with 0.5 M HCl (3 × 170 mL). The aqueous phase was extracted with dichloromethane (2 × 70 mL), the combined organic layers were dried over $MgSO_4$, filtered and the solvent was removed under reduced pressure. The crude product was purified by flash chromatography using dichloromethane/ethyl acetate (98:2) as eluent to afford **3** as a white solid (93% yield). ^{1}H-NMR (250 MHz, $CDCl_3$): δ = 8.11 (d, J = 9.8 Hz, 1H), 7.58 (d, J = 2.4 Hz, 1H), 7.10 (t, 1H), 6.93 (dd, J = 2.4, 1.0 Hz, 1H), 6.26 (d, J = 9.8 Hz, 1H), 4.49 (t, J = 5.8 Hz, 2H), 3.66 (t, J = 6.0 Hz, 2H), 2.21-1.93 (m, 4H) ppm; ^{13}C-NMR (62.9 MHz, $CDCl_3$): δ = 161.1, 158.2, 152.6, 148.6, 144.8, 139.1, 113.0, 112.6, 106.5, 105.0, 93.9, 71.9, 44.5, 29.1, 27.4 ppm; ESI-MS (ion trap): m/z: 293 $[M+H]^+$.

4-(4-iodobutoxy)-7H-furo[3,2-g]benzopyran-7-one **[PSBI]**. Compound **3** (1.7 mmol, 1 eq) and NaI (17 mmol, 10 eq) were added to anhydrous acetone (25 mL) under nitrogen. The suspension was stirred at 70 °C overnight. After addition of ethyl acetate (30 mL), the mixture was washed with deionized water (150 mL) and the organic phase was dried over $MgSO_4$. The crude product obtained after removal of the solvent under reduced pressure was purified by flash chromatography using dichloromethane/ethyl acetate (97:3) as eluent. Pure **PSBI** was obtained as a light yellow powder (80% yield). ^{1}H-NMR (250 MHz, $CDCl_3$): δ = 8.11 (d, J = 9.8 Hz, 1H), 7.58 (d, J = 2.4 Hz, 1H), 6.93 (dd, J = 2.4, 1.0 Hz, 1H), 6.26 (d, J = 9.8 Hz, 1H), 4.47 (t, J = 5.8 Hz, 2H), 3.29 (t, J = 6.5 Hz, 2H), 2.18 – 1.91 (m, 4H) ppm; ^{13}C-NMR (62.9 MHz, CDC13): δ = 161.1, 158.2, 152.6, 148.6, 144.8, 139.1, 113.0, 112.6, 106.5, 105.0, 93.9, 71.5, 30.8, 29.9, 5.9 ppm; ESI-MS (ion trap): m/z: 385 $[M+H]^+$.

Figure S2. Synthesis of P5TP from PSBI. Reagents and conditions: i) PPh_3 in toluene, 110°C, 15 h, N_2, 87%.

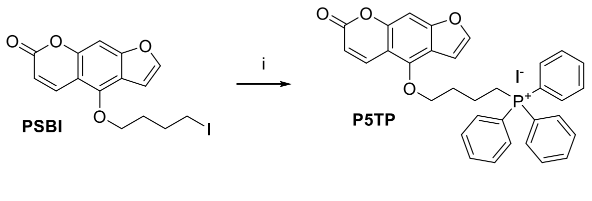

(4-((7-oxo-7H-furo[3,2-g]chromen-4-yl)oxy)butyl)triphenylphosphonium iodide **[P5TP]**. A mixture of **PSBI** (0.5 mmol, 1.0 eq) and PPh_3 (5.2 mmol, 10.5 eq) in HPLC-grade toluene (15 mL), was stirred and heated under nitrogen overnight at 120°C in darkness. The progress of the reaction was monitored by TLC. The mixture was then concentrated by stirring under vacuum and the residue was diluted in a minimal volume of DCM (2 mL) and precipitated with diethyl ether (150 mL). The solvent was decanted and the product was filtered under vacuum and washed with Et_2O (3 × 50 mL); residual solvent was removed under reduced pressure to afford **P5TP** as white powder (87% yield). ^{1}H-NMR (250 MHz, $CDCl_3$): δ = 7.98 – 7.61 (m; 16H), 7.56 (d, J = 2.4 Hz; 1H), 7.13 (dd, J = 2.4, 0.9 Hz; 1H), 7.03 (s; 1H), 6.12 (d, J = 9.8 Hz; 1H), 4.63 (t, J = 5.9 Hz; 2H), 3.94 (t, J = 14.7 Hz; 2H), 2.50 – 2.31 (m; 2H), 2.08 – 1.84 ppm (m; 2H); ^{13}C-NMR (62.9 MHz, $CDCl_3$): δ = 161.1 (CO), 158.2, 152.4, 148.6, 145.0, 143.0, 139.3, 135.1, 135.0 133.7, 133.5, 130.6, 130.4, 118.6, 117.2, 112.7, 112.1, 105.9, 105.7, 93.4, 71.2, 30.3, 30.0, 22.9, 22.1, 19.3, 19.2 ppm; ESI-MS (ion trap): m/z: 519 $[M]^+$.

Figure S3. Synthesis of PCTP from PSBI. Reagents and conditions: i) Cs_2CO_3 in DMF, hydroquinone, 45°C, 15 h, N_2, 84%; ii) 3-chloropropyl chloroformate in DCM, DMAP in THF, rt, 15 h, N_2, 94%; iii) NaI in acetone, 70°C, 15 h, N_2, 80%; iv) PPh_3 neat, 95°C, 3 h, N_2, 98%.

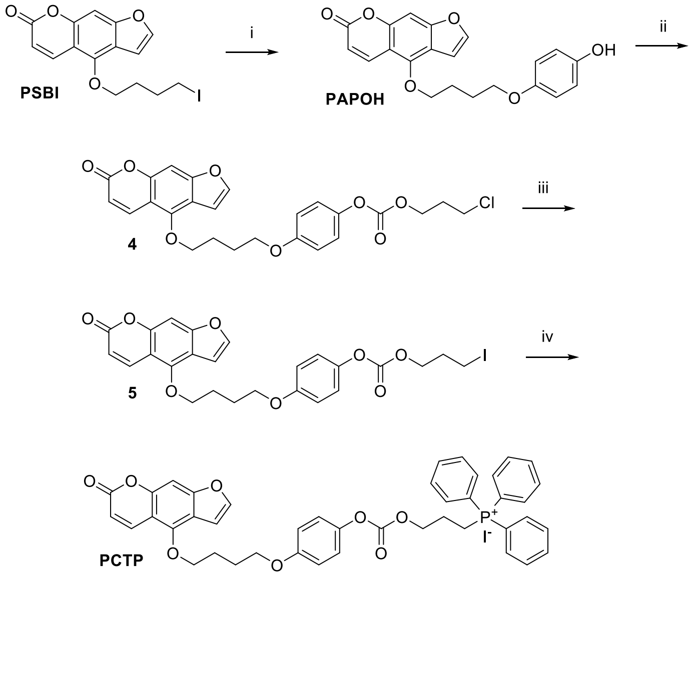

4-(4-(4-hydroxyphenoxy)butoxy)-7H-furo[3,2-g]benzopiren-7-one **[PAP-OH]**. Hydroquinone (4.5 mmol, 15 eq) was added under stirring to a mixture of **PSBI** (0.3 mmol, 1 eq) and Cs_2CO_3 (0.6 mmol, 2 eq) in DMF (15 mL). The mixture was allowed to react overnight in the dark and under stirring at 45 °C. After addition of ethyl acetate (90 mL), the mixture was extracted with 0.5 M aqueous HCl (5 × 50 mL). The organic phase was dried over $MgSO_4$, filtered and the solvent was removed under reduced pressure. The crude product was purified by flash chromatography using chloroform/acetone (90:10) as eluent. Pure **PAP-OH** was obtained as a cream-white solid (84% yield). ^{1}H-NMR (250 MHz, CDCl3): δ = 8.10 (d, J = 9.8, 1H), 7.75 (d, J = 2.4 Hz, 1H), 7.16 (dd, J

= 2.4, 1.0 Hz, 1H), 7.07 – 6.98 (m, 1H), 6.67 (m, 4H), 6.12 (d, J = 9.8 Hz, 1H), 4.55 (t, J = 6.0 Hz, 2H), 3.93 (t, J = 6.0 Hz, 2H), 2.08 – 1.80 (m, 4H) ppm; ^{13}C-NMR (62.9 MHz, CDCl3): δ = 160.8, 159.1, 153.7, 153.1, 152.2, 150.1, 146.2, 140.0, 116.6, 116.3, 113.9, 113.2, 107.2, 106.4, 93.8, 73.5, 68.6, 27.5, 26.7 ppm. ESI-MS (ion trap): m/z: 367 $[M+H]^+$.

3-chloropropyl (4-(4-((7-oxo-7H-furo[3,2-g]chromen-4-yl)oxy)butoxy)phenyl)carbonate **[4]**. **PAP-OH** (0.4 mmol, 1.0 eq) was dissolved in anhydrous THF (15 mL) with DMAP (0.5 mmol, 1.2 eq) at 0°C. After a few minutes, a solution of 3-chloropropyl chloroformate (0.4 mmol, 1.0 eq) in anhydrous dichloromethane (10 mL) was added dropwise to the reaction mixture. The system was stirred under an inert atmosphere overnight. Subsequently, EtOAc (100 mL) was added to the solution and then the mixture was extracted with 0.5 M HCl (5 × 70 mL). The organic phase was dried over $MgSO_4$ and filtered. The solvent was eliminated under reduced pressure and the crude product was purified by flash chromatography using DCM /EtOAc (97:3) as eluent to afford **4** as a white powder (94 % yield). ^{1}H NMR (500 MHz, $CDCl_3$) δ = 8.12 (d, J = 9.8 Hz, 1H), 7.57 (d, J = 2.3 Hz, 1H), 7.16 – 7.01 (m, 3H), 6.94 (d, J = 1.7 Hz, 1H), 6.87 (d, J = 9.0 Hz, 2H), 6.24 (d, J = 9.8 Hz, 1H), 4.53 (t, J = 5.9 Hz, 2H), 4.40 (t, J = 6.0 Hz, 2H), 4.05 (t, J = 5.7 Hz, 2H), 3.68 (t, J = 6.3 Hz, 2H), 2.20 (p, J = 6.2 Hz, 2H), 2.14 – 1.96 (m, 4H) ppm. ^{13}C NMR (126 MHz, $CDCl_3$) δ = 161.3, 158.4, 156.8, 154.0, 152.8, 149.0, 145.0, 144.9, 139.3, 122.0, 115.1, 113.3, 112.7, 106.8, 105.2, 94.0, 72.6, 67.8, 65.4, 40.9, 31.6, 27.0, 26.0 ppm. ESI-MS (ion trap): m/z 487 $[M+H]^+$.

3-iodopropyl (4-(4-((7-oxo-7H-furo[3,2-g]chromen-4-yl)oxy)butoxy)phenyl) carbonate **[5].** Compound **4** (3.8 mmol, 1.0 eq) was dissolved in anhydrous acetone (30 mL) saturated with NaI under nitrogen. The solution was stirred and heated at 70 °C in darkness overnight. EtOAc (100 mL) was then added to the solution and the mixture was washed with water (4 × 75 mL). The organic phase was dried over $MgSO_4$ and filtered. The solvent was evaporated under reduced pressure and the crude product was purified by flash chromatography using DCM/EtOAc (97:3) as eluent to afford **5** as a light yellow powder (80 % yield). ^{1}H NMR (300 MHz, $CDCl_3$) δ = 8.09 (d, J = 9.8 Hz, 1H), 7.56 (d, J = 2.4 Hz, 1H), 7.17 – 7.01 (m, 3H), 6.93 (d, J = 2.4 Hz, 1H), 6.89 – 6.80 (m, 2H), 6.22 (d, J = 9.8 Hz, 1H), 4.51 (t, J = 5.8 Hz, 2H), 4.30 (t, J = 6.0 Hz, 2H), 4.04 (t, J = 5.6 Hz, 2H), 3.27 (t, J = 6.8 Hz, 2H), 2.23 (p, J = 6.4 Hz, 2H), 2.14 – 1.92 (m, 4H) ppm. ^{13}C NMR (75 MHz, $CDCl_3$) δ = 161.2, 158.3, 156.7, 153.9, 152.7, 148.9, 144.9, 144.8, 139.3, 121.9, 115.1, 113.2, 112.6, 106.7, 105.2, 93.9, 72.5, 68.2, 67.8, 32.3, 27.0, 25.9, 1.0 ppm. ESI-MS (ion trap): m/z 579 $[M+H]^+$.

(3-(((4-(4-((7-oxo-7H-furo[3,2-g]chromen-4-yl)oxy)butoxy)phenoxy)carbonyl)oxy) propyl) triphenyl phosphonium iodide **[PCTP]**. A mixture (neat) of **5** (0.3 mmol, 1.0 eq) and PPh_3 (6.0 mmol, 20 eq), was mixed and heated under nitrogen at 95°C in darkness for 3 hours. After this time, the mixture was dissolved in DCM (5 mL) and the solute was precipitated with diethyl ether (150 mL). The solvent was decanted and the product was filtered under vacuum and washed with Et_2O (5 × 15 mL); residual solvent was removed under reduced pressure to afford **PCTP** as a light yellow

powder (98% yield). ^{1}H NMR (300 MHz, $CDCl_3$) δ = 8.08 (d, J = 9.8 Hz, 1H), 7.91 – 7.60 (m, 15H), 7.56 (d, J = 2.4 Hz, 1H), 7.05 (s, 1H), 7.00 (d, J = 9.0 Hz, 2H), 6.94 (d, J = 2.4 Hz, 1H), 6.81 (d, J = 9.1 Hz, 2H), 6.18 (d, J = 9.8 Hz, 1H), 4.52 (q, J = 6.0 Hz, 4H), 4.01 (t, J = 5.6 Hz, 2H), 3.87 (td, J = 13.3, 8.0 Hz, 2H), 2.29 – 1.77 (m, 6H) ppm. ^{13}C NMR (75 MHz, $CDCl_3$) δ = 161.3, 158.3, 156.7, 153.5, 152.6, 148.9, 145.0, 144.6, 139.4, 135.4, 133.8, 133.7, 130.8, 130.6, 121.9, 118.2, 117.1, 115.1, 113.2, 112.4, 106.6, 105.2, 93.8, 72.5, 67.8, 42.9, 26.9, 25.9, 22.3, 20.4, 19.7 ppm. ESI-MS (ion trap): m/z 713 $[M]^+$.
